# A Countermeasure Strategy against Peramine Developed by *Chilesia rudis* in the Endophyte–Ryegrass–Herbivore Model

**DOI:** 10.3390/jof10080512

**Published:** 2024-07-23

**Authors:** Manuel Chacón-Fuentes, Daniel Martínez-Cisterna, Marcelo Lizama, Valeria Asencio-Cancino, Ignacio Matamala, Leonardo Bardehle

**Affiliations:** 1 Agriaquaculture Nutritional Genomic Center, CGNA, Las Heras 350, Temuco 4780000, Chile; 2 Centro de Investigación Biotecnológica Aplicada al Medio Ambiente (CIBAMA), Universidad de La Frontera, Av. Francisco Salazar 01145, Casilla 54-D, Temuco 4811230, Chile; d.martinez11@ufromail.cl; 3 Programa de Doctorado en Ciencias de Recursos Naturales, Universidad de La Frontera, s/n, P.O. Box 58-D, Temuco 4780000, Chile; 4 Programa de Doctorado en Ciencias Agroalimentarias y Medioambiente, Facultad de Ciencias Agropecuarias y Medioambiente, Universidad de La Frontera, Av. Francisco Salazar 01145, Casilla 54-D, Temuco 4811230, Chile; 5 Escuela de Universidad de Valladolid Doctorado (ESDUVa), Departamento Producción Vegetal y Recursos Forestales, Escuela Técnica Superior de Ingenierías Agrarias, Campus “La Yutera”, Avda. de Madrid, 50, 34004 Palencia, Spain; 6 Carrera de Ingenieria en Recursos Naturales, Facultad de Ciencias Agropecuarias y Medioambiente, Universidad de La Frontera, Temuco 4811230, Chile; 7 Departamento de Producción Agropecuaria, Facultad de Ciencias Agropecuarias y Medioambiente, Universidad de La Frontera, Temuco 4811230, Chile

**Keywords:** native herbivore, detoxification, alkaloids, fungus, Erebidae, Epichloë

## Abstract

Exploitation of the symbiotic relationship between endophytic fungi and ryegrass is a crucial technique for reducing the incidence of insect pests. This is primarily due to the production of alkaloids, such as peramine, by the fungi. This alkaloid has been reported as both a deterrent and toxic to a variety of insects. However, insects have developed various strategies to counteract plant defenses. One of the most studied methods is their ability to sequester toxic compounds from plants. In this study, we examined the feeding preferences and adaptation to peramine in *Chilesia rudis*, a native Chilean larva. Using a no-choice assay, we assessed larval feeding preferences and mass gain on seven experimental lines and two commercial cultivars of endophyte-infected and non-infected ryegrass. Pupal development time and adult performance were evaluated post-assay. Additionally, we measured peramine content in larval carcasses, feces, and ryegrass leaves. Jumbo was the most preferred cultivar with 32 mm^2^ of leaf tissues consumed. The longest pupal development time was observed in L161 and ALTO AR1, both at 28 days. Wing length in adults was greatest in the Jumbo and L163 cultivars, measuring 1.25 cm and 1.32 cm, respectively. Peramine concentrations were detected in the bodies of *C. rudis*. In conclusion, this larva can adapt to endophyte-infected ryegrass and develop counter-adaptation mechanisms to mitigate the effects of peramine.

## 1. Introduction

Due to their sessile nature, plants cannot flee from stress and hence have developed sophisticated strategies to survive both biotic and abiotic stresses [1]. Plants have developed various defense mechanisms, such as producing toxic secondary metabolites, to defend themselves against herbivores and pathogens, minimizing damage and preserving their overall fitness. Oftentimes, secondary metabolites from a single class of compounds, e.g., alkaloids, occur in complex mixtures within plant tissues [2,3]. Alkaloids are defensive substances synthesized by plants to cope with attacks from organisms, animals, or insects. Alkaloids have been shown to influence the feeding behavior of insect herbivores in a variety of ways, acting as attractants, phagostimulants, deterrents, or repellents [4,5]. However, chemical defense strategies are never 100% effective, as insects have developed several ways to cope with the presence of secondary metabolites produced by their host plants. One such strategy is sequestration, which involves the selective uptake, transport, modification, storage, and deployment of plant secondary metabolites for the insect’s own defense. These strategies result from a counter-adaptation process generated by the coevolution between plants and their herbivores [6].

Fungal organisms known as endophytes can inhabit their host plants without causing damage. Endophytic fungi have been reported to increase yield production, improve nutrient uptake from the soil, and enhance the ability to cope with insect pests in *Lolium perenne* [7]. Four families of natural alkaloids are produced as a result of this symbiosis: ergot alkaloids, pyrrolizidines (lolines), indole-diterpenes (lolitrems), and pyrrolopyrazine (peramine). Individual alkaloids within these classes often have specific toxic effects depending on the type of herbivore (i.e., vertebrate vs. invertebrate). Lolines and peramine are well known for their insecticidal or insect-deterring effects [8,9]. Peramine functions exclusively as an anti-invertebrate defensive metabolite; it is not acutely toxic or a contact poison but deters feeding in several insect species [10], for example, the Argentine stem weevil (*Listronotus bonariensis*, Coleoptera: Curculionidae) [11]. Rowan [12] showed that peramine at concentrations from 0.1 to 10 μg/g deters both larvae and adults of the Argentine stem weevil, *L. bonariensis*. This symbiotic interaction between ryegrass and endophytes has also been associated with the control of the fall armyworm (*Spodoptera frugiperda*, Lepidoptera: Noctuidae); black cutworm (*Agrotis ipsilon*, Lepidoptera: Noctuidae); green bug (*Chinavia hilaris*, Hemiptera: Pentatomidae) and bluegrass billbug (*Stenophorus parvulus*, Coleoptera: Curculionidae), these being among the most relevant [13,14,15]. Additionally, peramine is distributed throughout all plant fluids and is water-soluble, offering protection to the entire plant, not just the areas colonized by the endophyte. Recent findings show that this alkaloid can accumulate in higher trophic levels, such as in predaceous ladybird beetles (*Adalia bipunctata* or *Harmonia axyridis*, Coleoptera: Coccinellidae) that feed on aphids reared on endophyte-infected grass [16]. Furthermore, Parra et al. [17] reported that extracts of feces containing peramine from animals fed on pastures infected with endophyte fungus increased the larval mortality (90%) of horn flies (*Haematobia irritans*, Diptera: Muscidae), reducing their survival compared to a control group fed on endophyte-free pastures.

The use of alkaloids to combat insects can trigger an arms race between plants and their herbivores. Plants evolve defense mechanisms, and insects develop countermeasures such as detoxification, sequestration mechanisms and passive accumulation [18,19]. This adaptation and co-evolutionary process suggests that insects can detoxify toxic metabolites, allowing them to cope with the defensive systems of their host plants. According to Nishida [20] and Opitz and Müller [21], these chemical compounds are diverse and are used by various lepidopterans for processes such as host selection, defense, and even mating. For example, species in the Papilionidae family have been associated with plants containing aristolochic acids, which are sequestered by larvae and transferred to eggs by adults, providing defense against predators [22]. Additionally, the genus *Battus* (Lepidoptera: Papilionidae) has been studied for its ability to use these compounds for host selection and defense [23,24]. Particularly, the Erebidae family has been studied in regard to some sequestration mechanisms of plant chemical defense. Nevertheless, there is no report about the storage, sequestration or detoxification mechanism of peramine by lepidopterans Erebidae.

In this context, *Chilesia rudis* (Lepidoptera: Erebidae) is a native, polyphagous, and univoltine larva from Chile, known as one of the most serious pests of grasslands due to its severe defoliating behavior [25,26,27]. In its larval stage, this insect is a severe and abundant defoliator of various plants, including introduced pastures grasses, ornamental plants, and native species such as *Ugni molinae* [28]. Previous studies by Nickisch-Rosenegk and Wink [29] have shown that pyrrolizidine alkaloids are sequestered by the larvae and adults of six European erebid moth species (*Spilosoma lubricipeda*, *Arctia caja*, *Phragmatobia fuliginosa*, *Callimorpha dominula*, *Diacrisia sannio*, and *Tyria jacobaeae*). Therefore, the main objective of this research was to evaluate the production of peramine due to endophyte fungus infection in seven experimental lines and two cultivars of ryegrass and to analyze the feeding preference and countermeasures to peramine of the native herbivorous *C. rudis*.

## 2. Materials and Methods

### 2.1. Plant Material

Ryegrass samples, including seven experimental lines (LE161, LE162, LE163, LE164, LE165, LE166, and LE167), as well as the commercial cultivars ALTO AR1 (endophyte-infected, denoted as E+) and JUMBO (endophyte-free, denoted as E−), were sourced from the plots of *L. perenne* at the Maquehue Experimental Station (University of La Frontera), located in Temuco, Chile [7]. During early summer (December 2023), tillers were manually harvested and carefully placed into paper bags for transportation to the Chemical Ecology Laboratory at the University of La Frontera. Upon arrival, leaves designated for bioassays were promptly utilized, while those earmarked for chemical analyses were stored in bags at −20 °C until subjected to analysis using high-performance liquid chromatography (HPLC).

### 2.2. Larval Rearing

The original population of *Chilesia rudis* larvae (30–40 mm) was collected from natural grassland in Temuco in 2020 [28]. Once the parental population was obtained, it was allowed to develop in acrylic boxes (30 × 30 × 30 cm) with cloth on each side to allow ventilation. The larvae were raised on the general-purpose lepidopteran diet (soy-wheat germ diet with vitamin and agar) (Frontier, agricultural Sciences, Newark, DE, USA) in 30 mL plastic cups. The cups were kept with a photoperiod of 16:8 (L:D), 60% R.H. and a temperature of 25 °C [8,30]. Briefly, 19 g of Agar was added to 875 mL of cold water. The mixture was brought to a full boil for 1 min with occasional stirring. Subsequently, the agar solution was transferred to the blender container, and the Dry Mix containing 144 g of ingredients including sucrose, soy flour (50%), stabilized wheat germ, salt mix, USDA vitamin premix, fiber, sorbic acid, methyl paraben, and ascorbic acid, was added. The mixture was blended for approximately 30 s or until thoroughly mixed. Finally, the prepared diet was dispensed immediately, and any unused diet was stored in the refrigerator for later use. The first generation of *C. rudis* (2021) was kept in rearing throughout its generation, which gave way to the offspring or F2 generation (2022), which were again raised in the same way as the parental population. This F2 population gave rise to the F3 population (2023), with which the experiments were conducted. For all experiments, larvae in their final instar were used.

### 2.3. Evaluation, Isolation and Characterization of Endophytic Fungus in Lolium perenne

The endophyte infection levels of each experimental line and cultivar were assessed using the method described by Dombrowski et al. [31] and Chacón-Fuentes et al. [32] prior to commencing the experiments. In summary, 120 tillers were harvested per cultivar or experimental line and the inner epidermis of a leaf sheath was carefully peeled off and mounted on a glass slide. Two drops of rose Bengal stain (Merck KGaA, Darmstadt, Germany) were applied to each sample and covered with a cover slip after 1–2 min. Subsequently, microscopic examination (40×) was conducted to determine the presence or absence of typical fungal mycelium, from which the infection percentage was calculated. Positive endophyte infection was determined by the presence of fungal hyphae in the assessed tillers. For endophyte isolation, *L. perenne* tillers underwent disinfection using 3% sodium hypochlorite, followed by rinsing with sterile distilled water. The tillers were then cultured on potato dextrose agar (PDA) supplemented with penicillin and streptomycin (100 µg g^−1^) for 3 weeks at 23 °C. *Epichloë* sp. presence in *L. perenne* was confirmed through molecular techniques following the methodology proposed by Chacón-Fuentes et al. [7]. Fungal tissue from petri dishes containing strains cultured from *L. perenne* was macerated under sterile conditions with liquid nitrogen. The obtained macerate was processed using the NucleoSpin Soil DNA isolation Kit (Macherey-Nagel) to extract non-denatured DNA, with purity index values analyzed using Gen5. Subsequently, a PCR reaction was conducted to associate the obtained DNA with the *Epichloë* sp. fungus identity.

### 2.4. No-Choice Assay

This experiment evaluated the consumption of *C. rudis* on seven experimental lines and two cultivars of ryegrass. A single *C. rudis* larva was placed in a petri dish (94 × 16 mm) with a hole in the top covered with mesh for air interchange. Dishes contained one fresh fully expanded leaf (10 cm wide × 1 cm long) per treatment (each treatment corresponded to each single experimental line or cultivar). The consumption was evaluated by scanning the leaves and calculating the foliar area removed by the larva with the software ImageJ (version 1.42), the ryegrass leaves were replaced every 12 h [28]. Furthermore, weight gain of the larvae was measured at the end of the experiment and calculated according to the following formula: Wf − Wi, where Wf is final weight and Wi corresponds to the initial weight. The bioassay was evaluated every 12 h over 7 days. At 7 days, the experiment was concluded. All larvae remained in the final instar without showing any evidence of molting or entering the pupal stage. Twenty replicates were carried out for each of the nine treatments.

### 2.5. Pupal Development Time and Adult Performance Evaluation

To assess the impact of peramine on the performance of *C. rudis,* the pupal development time was evaluated, and the same individuals from the no-choice assays were utilized. Briefly, pupal development time was determined by calculating the number of days from larvae to pupae (from original Petri dish). Furthermore, upon emergence, adult *C. rudis* were evaluated for various parameters: (1) total length of adults (cm), (2) male wing length (cm), and female wing length of adults, and (3) total weight of the last larval instar (g). Measurements were taken using a digital caliper and an analytical balance following the methodology outlined by Wang et al. [33].

### 2.6. Carcasses, Feces and Guts Peramine Extraction

In parallel, a new group of *C. rudis* larvae (20 larvae per experimental line or cultivar) were fed with leaves from the two commercial cultivars and the seven experimental lines separately. These larvae were allowed to feed, and upon reaching their final larval stage, they were sacrificed. Larvae were deprived of food for 3 days and dissected. The guts of the larvae were removed carefully using a scalpel (N° 11) under an optical glass to exclude any influence of remaining plant material. The carcasses of the larvae were stored in a paper bag and placed in an oven at 40 °C over three days or until it reached a constant weight. The insect material was ground to obtain a dry powder. A total of 25 mg of this material was placed in an amber Eppendorf tube (2 mL) and 1 mL of a mixture of methanol/water/formic acid (70:29.5:0.5 *v*/*v*) (chromatographic grade, Sigma-Aldrich, St. Louis, MO, USA) was added. Then, tubes were centrifuged at 6000 rpm for 3 min. The supernatant was collected in an amber chromatographic vial (1.7 mL) and analyzed by HPLC. Feces of the insects were collected each day and stored in the dark at −20 °C until chemical analyses by HPLC. These samples were freeze-dried for 24 h and then ground following the process explained above for larval carcasses. The guts obtained from the larval carcass protocol were also subjected to the same larval tissue extraction to determine if there was any remaining peramine inside them. Finally, the extracts of carcasses, feces and guts were filtered through 0.22 μm membranes before their injection into the HPLC [34].

### 2.7. Plant Material Extraction

Leaves of the seven experimental lines and the two commercial cultivars of *L. perenne* were dried in an oven for 3 days at 60 °C. Then, the samples were milled in a grinder until they reached a particle size of 2 mm and placed in a polypropylene tube in darkness. Next, 0.5 g of milled material was first extracted with 3 mL of methanol and then with 3 mL of chloroform (HPLC grade, Sigma-Aldrich, St. Louis, MO, USA) for 30 min at room temperature. Subsequently, 3 mL of hexane and 3 mL of water (HPLC grade, Sigma-Aldrich, St. Louis, MO, USA) were added, and the mixture was centrifuged at 6000× *g* for 5 min. Afterward, 1 mL of the aqueous phase was passed through a CCX column (UCT 2731 Bartram Road, Bristol, PA, USA). In the same column, 1 mL of HPLC water and finally 0.5 mL of 0.5% formic acid were used to elute the peramine from the column. Samples were collected in amber vials, filtered through a 0.22 μm membrane, and analyzed by HPLC [28].

### 2.8. HPLC-DAD Analysis

Twenty microliters of samples were injected into a Shimadzu HPLC system (LC-20A Prominence, Kyoto, Japan) equipped with a C-18 column (250 × 4.6 mm I.D.; particle size 5 μm) maintained at 25 °C. A peramine standard (purchased from AgResearch, Hamilton, New Zealand) was monitored at 280 nm. The analysis was conducted using an isocratic mobile phase composed of 80% guanidinium buffer (A) and 20% acetonitrile (B) at a flow rate of 1 mL/min, with each HPLC analysis completed in approximately 10 min. The mobile phase was prepared by dissolving 1.44 g of guanidinium carbonate in 800 mL of HPLC water, adding formic acid (1.3 mL/L), and 200 mL of HPLC-grade acetonitrile (Merck KGaA, Darmstadt, Germany). Samples were prepared in triplicate, and the amount of peramine was determined from a calibration curve generated by injecting commercial standards in a serial dilution from 100 ppm to 0.01 ppm [35].

### 2.9. Survival Rate Test

A new group of 20 larvae per experimental line or commercial cultivar was used to evaluate larval survival. Each larva was placed individually in a separate Petri dish containing a whole leaf from the endophyte-free cultivar Jumbo (10 × 1 cm). Subsequently, 100 μL of peramine at concentrations of 1, 5, 10, and 100 mg/L were added to each leaf. This method was replicated twenty times for each of the seven experimental lines and two commercial cultivars. Then, the larvae were allowed to feed for two days, with the leaves being replaced every 12 h. After the two-day period, mortality was assessed by counting the deceased individuals, and a percentage was calculated based on the total number of surviving individuals versus those that died [36].

### 2.10. Statistical Analysis

The statistical software Statistix 10 (Tallahassee, Florida) was utilized to analyze the presence of endophyte in the seven experimental lines and two commercial cultivars, as well as the effect of peramine on no-choice assays (leaf consumption), pupal development time, adult performance, survival rate tests, and the presence of peramine in leaves, carcasses, feces, and guts of *C. rudis*. Briefly, to analyze the presence of endophytic fungi, a chi-square test was conducted for each experimental line or commercial cultivar. In addition, an ANOVA followed by a Bonferroni test was conducted to analyze differences in the presence of endophyte among the different experimental lines and cultivars. To analyze the no-choice assays, survival rate range, and evaluation of morphological characteristics, as well as peramine content, an analysis of variance (ANOVA) followed by Tukey’s multiple comparison test was conducted. Additionally, bivariate Pearson correlations were calculated to estimate specific relationships among variables. Values of *p* ≤ 0.05 were considered significant. Results are presented as means with their corresponding standard errors.

## 3. Results

### 3.1. Evaluation, Isolation and Characterization of Endophytic Fungus in Lolium perenne

The experimental results presented in Table 1 depict the endophyte infection rates across various line experimental cultivars along with JUMBO (E−) and ALTO AR1 (E+). Among the experimental lines, L161 and ALTO AR1 showcased complete endophyte colonization, with 100% infection rates, whereas L162, L164, L166, and L165 exhibited significant endophyte presence, ranging from 74% to 91.6%. Conversely, L163 and L167 exhibited lower endophyte colonization rates, with values of 10% and 11%, respectively. As expected, JUMBO (E−) showed no endophyte colonization, while ALTO AR1 (E+) showed complete colonization, mirroring the findings observed in L161 (*F*_8,104_ = 2.35, *p* < 0.001).

Table 2 provides the results of a PCR analysis specifically targeting the Tub2 intron to detect the presence of the endophytic fungus *Epichloë* sp. The results indicate the confirmed presence or absence of the endophytic fungus in the analyzed samples. The columns “IS-RS-5′; IS-NS3” and “IS-tub2w-5′; IS-tub2w-3” represent the testing conditions used. In the column corresponding to “IS-RS-5′ and IS-NS3” as well as in “IS-tub2w-5′ and IS-tub2w-3”, all samples, including experimental lines L161 to L167, as well as the commercial cultivar ALTO AR1 (E+), show the confirmed presence of the endophytic fungus, indicated by the symbol “+”. Meanwhile, the commercial cultivar JUMBO (E−) exhibits the absence of the endophytic fungus.

### 3.2. No-Choice Assay

**Figure 1:**
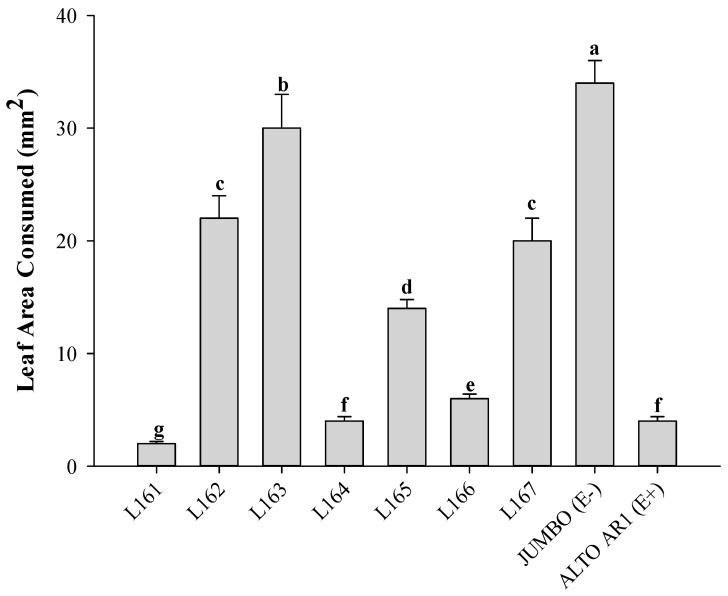
Leaf area consumed from the seven experimental lines and two cultivars of ryegrass by *C. rudis* in a no-choice test. Different letters indicate significant differences according to Tukey’s test (*p* ≤ 0.05). The data correspond to the mean with its corresponding standard error.

Leaf area consumed from the seven experimental lines by *C. rudis* larvae on grasses, measured in cm^2^ in a no-choice assay, reveals significant variations among different experimental lines (Figure 1). Overall, the commercial cultivar JUMBO (E−) exhibits the highest level of consumption, averaging 34 ± 2.0 mm^2^ (leaf-removing area), closely followed by the experimental line L163 with 30 ± 3.0 mm^2^, while the commercial cultivar ALTO AR1 (E+) and the experimental line L164 record the lowest consumption levels, averaging 4 ± 0.4 mm^2^ each (*F*_8,1011_ = 17.25, *p* < 0.001). These discrepancies underscore the importance of different experimental lines in grass susceptibility to larval performance and suggest the need for further investigation to understand the contributing factors to these variations.

### 3.3. Pupal Development Time and Adult Performance Evaluation and HPLC-DAD Analysis

Table 3 shows the relationship between peramine in leaves and several analyses of larvae performance and content of peramine in tissues of larvae. For example, when analyzing the pupal development time (*F*_8,171_ = 19.91, *p* < 0.001) and peramine concentration in leaves (*F*_8,18_ = 1377, *p* < 0.001), it is observed that larvae feeding from leaves from experimental lines and the commercial cultivar with lower peramine concentration in the leaves, such as JUMBO (E−) (0.0 ± 0.0µg/g), have a shorter pupal development time (23 ± 1.4 days), while larvae feeding from leaves from experimental lines or the commercial cultivar with a higher concentration, such as ALTO AR1 (E+) (184.1 ± 29.8 µg/g), have a longer pupal development time (28 ± 1.2 days). This relationship suggests that the concentration of peramine might be related to the pupal development time, resulting in an increase of up to 5 days in the larva-to-pupa cycle for this species when it is subjected to a diet with the presence of peramine (ALTO AR1 (E+)). In regard to the amount of peramine in leaves related to the length of *C. rudis* larvae, it is observed that experimental lines and commercial cultivar with higher concentrations of peramine in the leaves, such as L161 (179.3 ± 24.5 µg/g) and ALTO AR1 (E+) (184.1 ± 29.8 µg/g), show significantly smaller larvae length than other experimental lines such as L163 and L167 as well as the commercial cultivar JUMBO (E−) (*F*_8,171_ = 142.8, *p* < 0.001). For example, L163 has a larval length of 2.9 ± 0.2 cm, L167 has a length of 3.1 ± 0.1 cm, and JUMBO (E−) has a length of 3.2 ± 0.3 cm, while ALTO AR1 (E+) has a length of 1.4 ± 0.1 cm. This observation suggests that the concentration of peramine in the leaves has a direct relationship with the decrease in larval length. Furthermore, the weight of the larva decreases as the amount of peramine increases, with the lowest amount of peramine (JUMBO (E−)) corresponding to a higher larval weight (0.7 ± 0.1 g) and the highest amount of peramine.

ALTO AR1 (E+) corresponded to a lower larval weight (0.3 ± 0.0 g). On the other hand, for both male (*F*_8,135_ = 63.1, *p* < 0.001) and female (*F*_8,27_ = 16.55, *p* < 0.001) adults, there was a trend of decreasing wing length with an increase in the amount of peramine, with the lowest amount of peramine (JUMBO (E−)) corresponding to longer wing lengths and the highest amount of peramine (ALTO AR1 (E+)) corresponding to shorter wing lengths. In addition, when comparing the concentration of peramine in leaves with that found in the carcasses, feces, and guts of the larvae, a general trend was observed where experimental lines and commercial cultivars with higher concentrations of peramine in the leaves also tended to have higher concentrations in carcasses, feces, and guts (*F*_8,171_ = 981.1, *p* < 0.001; *F*_8,171_ = 20,062, *p* < 0.001; *F*_8,171_ = 2484 *p* < 0.001, respectively). For example, the experimental line L161, with a high concentration of peramine in the leaves (179.3 ± 24.5 µg/g), also shows a high concentration in the carcass (12.1 ± 1.0 µg/g) and feces (31.7 ± 4.2 µg/g), contrary to what happens in other experimental lines such as L167, which presents 17.1 ± 6.1 µg/g of peramine in leaves and therefore the concentration of this alkaloid in the carcass and feces is 2.2 ± 0.2 µg/g and 1.1 ± 0.4 µg/g, respectively. Complementing this, the Pearson correlation revealed various correlations among the studied variables. For instance, a strong positive correlation was observed between the concentration of peramine in the leaves and its presence in the carcass and feces of the larvae, with correlation coefficients of 0.976 and 0.9937, respectively. Additionally, significant correlations were found between the concentration of peramine in the carcass and feces (0.9498), as well as between the concentration of peramine in feces and intestines (0.5086). On the other hand, notable negative correlations were found between total length and the concentration of peramine in leaves, carcasses, and feces, with correlation coefficients of −0.9128, −0.8348, and −0.8537, respectively. These correlations suggested both positive and negative relationships among the studied variables, indicating potential complex interactions in the biology and development of *Chilesia rudis* larvae. Finally, these observations suggest possible complex interactions between the concentration of peramine in the leaves and various aspects of the development and physiology of *Chilesia rudis*.

### 3.4. Survival Rate Test

**Figure 2:**
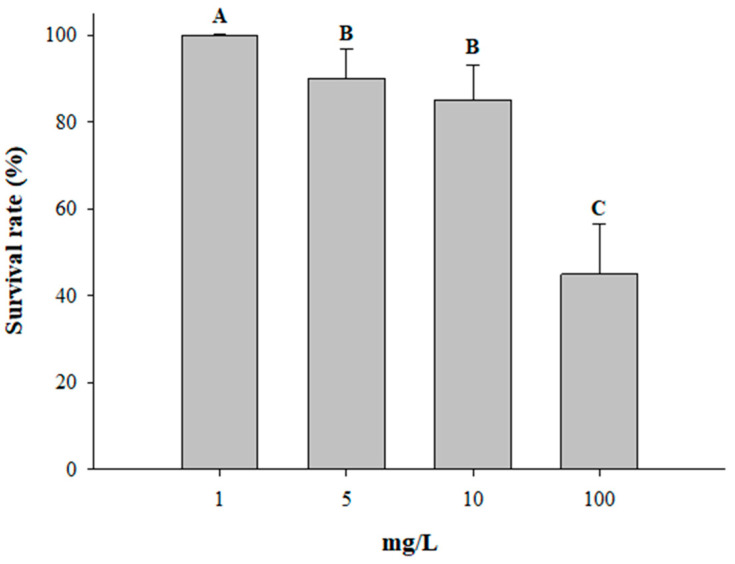
Survival rate for C. rudis larvae feeding on leaves of JUMBO (E−) subjected to several doses of peramine. Different letters indicate significant differences (*p* ≤ 0.05) based on ANOVA test following by Tukey’s test.

The results of the mortality assay on *C. rudis* larvae using different concentrations of peramine reveal that as the concentration of peramine increases, the percentage of larval survival decreases (Figure 2). The lowest concentration of 1 mg/L shows high survival rates of 100%, while the highest concentration of 100 mg/L results in the lowest survival rate, recording 45%. This pattern suggests a significant dose–response relationship, where higher concentrations of peramine correlate with greater larval mortality (*F*_3,76_ = 9.53, *p* < 0.001).

## 4. Discussion

The experimental findings outlined in the study provide a comprehensive analysis of the presence and infection rates of *Epichloë* sp. in *L. perenne* across various experimental lines and commercial cultivars. This incorporation of endophytic fungal infection in ryegrass has facilitated the control of several pests, such as *L. bonariensis*, due to specific strains that produce peramine. However, our results indicate that the concentration of peramine in ryegrass is not directly correlated with the percentage of endophyte infection. This discrepancy arises because various endophyte strains independently produce different groups of alkaloids. For example, Guerre [37] identified *Epichloë festucae* var. *lolii* as one of the endophytic fungi responsible for producing lolitrem B in ryegrass, while studies by Schardl et al. [38] reported various fungi capable of producing peramine, such as *N. gansuense* and *E. typhina*. The effects of these alkaloids generally relate to their toxicity, deterrence, or mortality on insects.

As plant defense mechanisms evolve, they also prompt insects to develop countermeasures [39]. Within this framework, our no-choice experiment results demonstrated that the cultivar JUMBO was more preferred by the herbivore *C. rudis* compared to other cultivars and experimental lines. Interestingly, despite this preference, larvae showed similar weight gain across all treatments. This suggests that while peramine may act as a deterrent, *C. rudis* might be either storing, sequestering, or passively accumulating this alkaloid to mitigate its toxicity. Chacón-Fuentes et al. [28] reported an example of potential detoxification, sequestration, or passive accumulation in *C. rudis.* They found that when larvae consumed artificial diets containing phenolic compounds like kaempferol, rutin, myricetin, and gallic acid at concentrations of 10 or 100 mg/g, it led to reduced consumption percentages and increased larval mass without affecting survival. This study underscores the role of sequestration and passive accumulation as significant defense strategies in herbivores, particularly within the Lepidoptera order [29,40,41,42].

Research has highlighted a link between the sequestration of specific compound classes and the level of dietary specialization observed in insects. For instance, our survival assays underscore the potential role of peramine as a potent deterrent against herbivory in ryegrass, highlighting its ecological significance in plant–insect interactions and suggesting its potential application in pest management strategies. However, this effect is observed at high concentrations. Interestingly, at concentrations ranging from 1 to 10 mg/L, mortality was not affected, consistent with findings reported by Chacón-Fuentes et al. [28], suggesting a possible passive accumulation of compounds by the larvae as an adaptation method to the alkaloids present in these grasses. In complement to our results, Lampert and Bowers [43] and Lampert et al. [44] compared the sequestration of iridoid glycosides between the specialist larva *Junonia coenia* and other herbivores (*Anartria jatrophae* and *Vanessa cardui*); the specialist sequestered nearly 12% more iridoid glycosides than other species. Therefore, this phenomenon has been identified as a result of evolutionary relationships between insects and phytochemicals [45].

In summary, our results indicate that the concentration of peramine in various experimental lines and cultivars of ryegrass ranged from 15.2 μg/g to 184.1 μg/g. These values are higher than concentrations reported by Spiering et al. [46], who found values ranging from 14 to 34 μg/g in various parts of ryegrass. Studies by Fuchs et al. [16] showed that ryegrass infected with endophytes produced concentrations of peramine around 0.06 μg/g, while their herbivore, *Rhapalosiphum padi*, was able to accumulate 0.19 μg/g in their tissues. Generally, the peramine content in ryegrass is influenced by endophyte strain, environmental factors, and host age [47]. The presence of fungus did not significantly affect the total length of insects, with Jumbo (E−) showing lengths of 3.2 ± 0.3 cm and Alto AR1 (80% endophyte) showing lengths of 1.40 ± 0.1 cm. Furthermore, insect mass was also not significantly affected when comparing cultivars JUMBO and Alto AR1. Our results suggest that the pyrrolopyrazine alkaloid peramine acted as a deterrent for *C. rudis* but was not toxic and did not significantly affect larval weight due to the ability of larvae to store peramine within their tissues and eliminate a percentage of the alkaloid through feces. Future research will focus on determining the ecological role of sequestered compounds or passive accumulation by *C. rudis* and evaluating other alkaloids that may be sequestered from ryegrass, such as PAs. Finally, these results offer insights into the variability of endophyte colonization among different experimental lines, indicating potential candidates for further investigation and breeding programs aimed at enhancing endophyte resistance in crops.

## Figures and Tables

**Table 1:** Percentage of endophyte fungal infection in tillers of the several experimental lines and commercial cultivars of ryegrass. Different letters indicate significant differences based on ANOVA followed by Bonferroni test. * mean significant differences into every experimental line or cultivars based on Chi-square test (*p* ≤ 0.05).

Line Experimental	N° of Plants Tested	(E+)	(E−)	Endophyte (%)
L161	120	120	0	100 *^a^
L162	120	89	31	74 *^d^
L163	120	12	108	10 *^e^
L164	120	110	10	91.6 *^b^
L165	120	100	20	83.3 *^c^
L166	120	110	10	91.6 *^b^
L167	120	13	107	11 *^e^
JUMBO (E−)	120	0	120	0 *^f^
ALTO AR1 (E+)	120	120	0	100 *^a^

**Table 2:** PCR analysis of Tub2 Intron for presence of *Epichloë* sp. endophyte. "+" means the presence of endophyte, while “−” means the absence of endophyte.

	PCR (Tub2 Intron)	
Experimental Lines or Commercial Cultivars	IS-RS-5′; IS-NS3′	IS-tub2w-5′; IS-tub2w-3′
L161	+	+
L162	+	+
L163	+	+
L164	+	+
L165	+	+
L166	+	+
L167	+	+
JUMBO (E−)	−	−
ALTO AR1 (E+)	+	+

**Table 3:** Peramine concentration in leaves, carcasses, feces and guts, pupal development time and performance of the adult of *C. rudis* after no-choice test for each experimental line and commercial cultivar of ryegrass. Different letters per column indicate significant differences (*p* ≤ 0.05) based on ANOVA test followed by Tukey’s test.

Experimental lines and Cultivars	Peramine in Leaves (µg/g)	Pupal Development Time (days)	Length (cm)	Weight (g)	Wing Length ♀ (cm)	Wing Length ♂ (cm)	Peramine in Carcasses (µg/g)	Peramine in Feces (µg/g)	Peramine in Guts (µg/g)
L161	179.3 ± 24.5 ^a^	28 ± 1.1 ^a^	1.7 ± 0.1 ^e^	0.5 ± 0.0 ^b^	0.17 ± 0.0 ^b^	0.63 ± 0.1 ^e^	12.1 ± 1.0 ^a^	31.7 ± 4.2 ^a^	0.0 ± 0.0 ^e^
L162	99.8 ± 17.3 ^d^	25 ± 1.2 ^b^	1.8 ± 0.1 ^d^	0.6 ± 0.1 ^b^	0.14 ± 0.0 ^d^	0.99 ± 0.1 ^c^	8.2 ± 0.9 ^c^	13.7 ± 2.7 ^d^	0.8 ± 0.1 ^c^
L163	15.2 ± 4.9 ^e^	24 ± 1.0 ^c^	2.9 ± 0.2 ^b^	0.7 ± 0.0 ^a^	0.13 ± 0.0 ^d^	1.32 ± 0.2 ^a^	1.6 ± 0.8 ^d^	1.1 ± 0.3 ^e^	0.0 ± 0.0 ^e^
L164	160.8 ± 39.9 ^b^	26 ± 2.1 ^b^	1.9 ± 0.2 ^d^	0.5 ± 0.0 ^b^	0.15 ± 0.0 ^c^	0.70 ± 0.1 ^d^	11.3 ± 2.1 ^a^	27.9 ± 4.2 ^b^	0.2 ± 0.0 ^d^
L165	127.0 ± 34.2 ^c^	25 ± 2.0 ^b^	2.4 ± 0.1 ^c^	0.6 ± 0.0 ^b^	0.16 ± 0.0 ^c^	1.19 ± 0.2 ^b^	9.5 ± 0.9 ^b^	19.7 ± 2.3 ^c^	1.5 ± 0.1 ^b^
L166	146.4 ± 21.2 ^c^	26 ± 1.5 ^b^	2.0 ± 0.1 ^d^	0.5 ± 0.1 ^b^	0.12 ± 0.0 ^e^	0.80 ± 0.1 ^d^	10.1 ± 0.1 ^b^	24.0 ± 2.1 ^b^	1.7 ± 0.1 ^a^
L167	17.1 ± 6.1 ^e^	24 ± 1.1 ^c^	3.1 ± 0.1 ^a^	0.8 ± 0.1 ^a^	0.15 ± 0.0 ^c^	1.20 ± 0.2 ^b^	2.2 ± 0.2 ^d^	1.1 ± 0.4 ^e^	0.0 ± 0.0 ^e^
JUMBO (E−)	0.0 ± 0.0 ^f^	23 ± 1.4 ^c^	3.2 ± 0.3 ^a^	0.7 ± 0.1 ^a^	0.20 ± 0.0 ^a^	1.25 ± 0.2 ^b^	0.0 ± 0.0 ^e^	0.0 ± 0.0 ^f^	0.0 ± 0.0 ^e^
ALTO AR1 (E+)	184.1 ± 29.8 ^a^	28 ± 1.2 ^a^	1.4 ± 0.1 ^f^	0.3 ± 0.0 ^c^	0.17 ± 0.0 ^b^	0.43 ± 0.1 ^f^	10.5 ± 1.1 ^a^	32.8 ± 4.4 ^a^	1.3 ± 0.1 ^b^

## Data Availability

Data are contained within the article.
